# Functional Ambulation Profile (FAP) Score as a Potential Marker of Gait Analysis in Myotonic Dystrophy Type 1

**DOI:** 10.3389/fneur.2020.00392

**Published:** 2020-05-19

**Authors:** Sunyoung Kim, Yong-Hyun Lim, Kyunghun Kang, Donghwi Park, Ho-Won Lee, Jin-Sung Park

**Affiliations:** ^1^Department of Neurology, University of Ulsan College of Medicine, Ulsan University Hospital, Ulsan, South Korea; ^2^Center of Self-Organizing Software-Platform, Kyungpook National University, Daegu, South Korea; ^3^Department of Neurology, School of Medicine, Kyungpook National University Chilgok Hospital, Kyungpook National University, Daegu, South Korea; ^4^Department of Physical Medicine and Rehabilitation, University of Ulsan College of Medicine, Ulsan University Hospital, Ulsan, South Korea

**Keywords:** myotonic dystrophy, gait, FAP score, gastrocnemius, tibialis anterior, instability

## Abstract

Recent studies on Myotonic dystrophy type 1 (DM1) have shown profound impairments in gait, leading to falls. We analyzed functional ambulation profile (FAP) score that reflects the temporal and spatial gait characteristics and investigated correlations with the lower limb muscle magnetic resonance imaging (MRI) and 6 min walk test (6MWT). Twenty patients with DM1 and 20 controls participated in this study. The 6MWT and gait analysis including FAP scores via GAITRite were performed in all patients and controls. DM1 patients displayed slower gait, shorter stride length, shorter stance length, and lower FAP score. Among lower extremity muscles, the gastrocnemius, soleus and tibialis anterior showed the most severe fat infiltration and these crural muscles significantly correlated with FAP and 6MWT. Among crural muscles, tibialis anterior was the most important muscle affecting gait speed, whereas the gastrocnemius contributed substantially to gait instability. FAP score correlated with the muscle imaging and 6MWT in DM1. Therefore, FAP score maybe used as an non-invasive marker that reflects deterioration of gait and a possible surrogate biomarker in DM1.

## Introduction

Myotonic dystrophy type I (DM1; Steinert disease; OMIM160900) is the most common form of adult onset muscular dystrophy. Classically, DM1 is characterized by myotonia and pronounced distal weakness in both upper and lower limbs (1). The proportion of wheelchair-bound DM1 patients is low as walking ability is generally maintained until the severe stage (2). However, DM1 is commonly associated with gait alterations that impair functional capacity, reduce quality of life, and lead to a dramatic increase in fall risk (3, 4). Only a limited number of studies have described gait impairments in patients with DM1, and different technical approaches were used by each study in small samples of patients (5–7). These studies using 3D motion analysis with electromyography and infrared-emitting diodes have indicated an important contribution of distal lower limb muscle weakness to gait impairments in DM1. However, muscle strength was not quantitatively assessed and it did not show weakness until moderate muscle degeneration. Therefore, the specific influence of muscle on gait abnormalities associated with DM1 remains unclear. Recent studies have shown the usefulness of muscle magnetic resonance imaging in identifying patterns of muscle involvement in various genetic myopathies. It also provides an excellent approach for measuring the progression of muscle disease in recent clinical trials (8, 9) The GAITRite is a portable gait analysis tool for automated measurement of spatiotemporal gait parameters (10). It is frequently used for clinical and research purposes, and has been validated with different patient groups such as for stroke (11), multiple sclerosis (12), Parkinson's disease (13, 14), Freidreich ataxia (15), and DM1 (16). Among the parameters in GAITRite, we focused on the FAP score that represent the quantitative spatiotemporal gait parameters that encompasses gait speed and distance that evaluates fall risks and gait balance as well. To the best of our knowledge, this is the first study to investigate the relationship among quantified and computerized gait assessment with emphasis on FAP score in relation to muscle imaging and 6MWT in DM1.

## Methods

### Participants

We included patients with DM1 from different families, and age- and sex-matched controls who attended the clinic in Department of Neurology at Kyungpook National University Chilgok Hospital from January 22, 2018 to December 6, 2018. This study was approved by the Institutional review board of Kyungpook National University Chilgok hospital (KNUCH 2018-10-019). Medical records of age, sex, duration of disease, and muscle weakness were reviewed. A diagnosis of DM1 was genetically confirmed, and patients who were not able to walk independently were excluded in this study. Lower muscle MRI evaluation and gait analysis were performed using GAITRite, a single layer pressure sensitive walkway measuring temporal and spatial parameters in patients and controls. Each participant was informed about the test procedure and participated in the study after providing written consent.

### MRI Acquisition

Muscle MRI was performed with a 3.0 Tesla scanner (*Discovery MR750, General Electric*, Milwaukee, WI) using the implemented quadrature body coil, 32-channel body array coil for radiofrequency (RF) remission as previously mentioned (17, 18). We graded the degree of fatty infiltration in the T1 weighted image of the individual muscles according to the Mercuri scale that is widely used in various muscle disease (19). The scale consists of a 5 grading system that range from 0 to 4. 0 represents normal appearance of the muscles and 4 is an end stage of the muscle with diffuse fatty infiltrations.

### Gait Assessment

#### Walking Endurance Test

The 6 minute walk test (6MWT) which is the most commonly used parameter used in evaluation of motor performance in various myopathies was utilized. 6MWT not only reflects the overall motor function but it also represents the cardiopulmonary status of the patients (20).

### GAITRite Procedures

**Figure 1:**
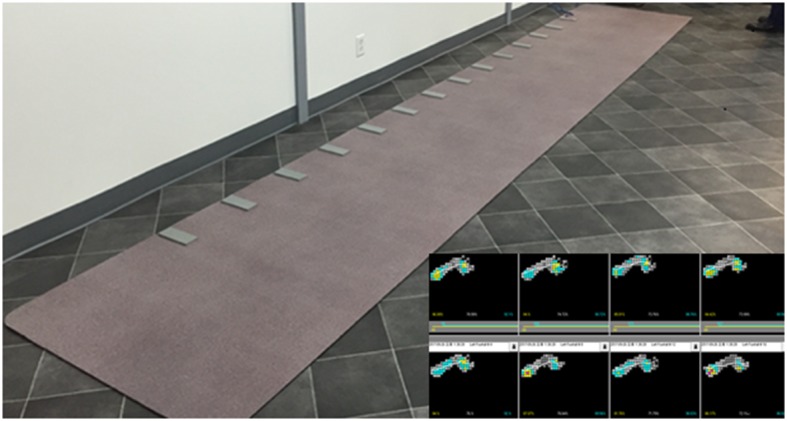
The GAITRite apparatus used in the study.

Gait assessment was conducted using a computer-based, pressure-activated carpet system with a sampling rate of 120 Hz (GAITRite, CIR System, Havertown, PA). The GAITRite is a 6-m long and 0.6-m wide mat instrumented with pressure-sensors that enable quantitative assessment of spatiotemporal parameters of gait (Figure 1). Data were collected by a series of on-board processors and transferred to a computer via an interface cable. The GAITRite software immediately transformed the raw data into spatiotemporal gait parameters. We recorded general parameters such as step count, velocity, cadence, and functional ambulation profile (FAP) score. The FAP score is an overall score based on the step length/extremity length ratio, step time, normalized velocity, and dynamic base of support (21). Temporal and spatial gait parameters were extracted for each individual foot. The temporal gait parameters included step time, stride time, swing time, stance time, and heel-off-on-time (the heel on time for the next foot minus the heel off time for the current foot). The spatial gait parameters included step length, stride length, step extremity ratio, and base of support.

### Test Procedures for Gait Analysis

Participants were instructed to walk barefoot and at a comfortable self-selected speed without physical assistances or any walking aids. The walking trial was repeated four times to obtain stable data. The mean values obtained from walking four times were used for the final analysis. To prevent acceleration and deceleration effects, participants started walking 1 m before reaching the active area of the electronic walkway and completed their walk 1 m beyond it. All participants were given breaks between walking trials as requested to avoid the onset of fatigue.

### Statistical Analysis

For gait analysis, we used a repeated-measures analysis of variance (ANOVA) with one within-subject factor (left/right foot) and one between-subject factor (patients with DM1/age-matched healthy controls). A paired *t*-test was used to compare patients' clinical data, such as age of onset, disease duration, CTG repeats, and general gait parameters (e.g., step count, distance, cadence, and FAP score) with those of controls. Spearman's correlation coefficient (rho) was used to assess the relationship between gait parameters and muscle MRI grading scores of different muscle groups. Statistically significant correlations (defined by two-tailed *p* < 0.05) were reported. Exact *p*-values were reported unless *p* < 0.01. All analyses were conducted in IBM SPSS v.24. Heat maps and hierarchical clustering were generated with the *ggplot2* and *ComplexHeatmap* packages of R software (version 3.6.1, www.r-project.org).

## Results

### Gait Parameters of DM1 Patients and Controls

In total, 20 patients with DM1 were included. The mean age of onset was 44 ± 9.7 years. The mean disease duration was 13.4 ± 7.30 years. The mean number of CTG repeats was 369.05 ± 211.66 (Table 1). In total, 20 controls were included (mean age, 51.30 ± 4.71 years; 7 males). For gait parameters, an initial comparison between right and left lower limbs was made. As no significant difference between the two limbs was detected, the means from both sides were pooled. The step count reflects the total number of steps counted within a walkway and cadence reflects the total number of steps within a minute. In general, our results in DM1 patients had a higher step count score (*p* < 0.001), slower velocity (*p* < 0.001), and shorter cadence (*p* < 0.01) than those of controls. The FAP score was also significantly lower in DM patients than in controls (*p* < 0.05) (Table 3). For spatio-temporal gait parameters, DM1 patients showed shorter step length (*p* < 0.001), shorter stride length (*p* < 0.001), lower step extremity ratio (*p* < 0.001), wider base of support (*p* < 0.001), longer step time (*p* = 0.001), longer stride time (*p* = 0.001), longer stance time (*p* < 0.001), and shorter heel off heel on strike time (*p* < 0.001) compared with those of age and sex matched controls (Table 2).

**Table 1:** Baseline characteristics of DM1 patients.

	**Patients with myotonic dystrophy**
Number (M:F)	20 (11: 9)
Age (year)	42.65 ± 9.66
Age Onset (year)	44.45 ± 9.7
Disease duration (year)	13.4 ± 7.30
CTG repeat number	369.05 ± 211.66
CK level	259.1 ± 125.7
6MWT	337.75 ± 86.95

*6MWT, 6 min walking test; M, male; F, female; CK, creatine kinase*.

**Table 2:** The spatio-temporal gait parameters from GAITRite difference between DM1 and controls.

**Spatio-temporal gait parameters**	**Patients with myotonic dystrophy**	**Controls**	***p***
Step count	35.4 ± 8.57	26.5 ± 3.5	<0.001
Velocity (m/sec)	0.89 ± 0.19	1.25 ± 0.19	<0.001
Cadence (steps/min)	107.22 ± 17.53	121.03 ± 9.59	<0.01
FAP score	89.3 ± 10.57	94.9 ± 5.76	<0.05
Step time (sec)	0.57 ± 0.02	0.50 ± 0.02	0.001
Stride time (sec)	1.14 ± 0.03	0.99 ± 0.03	0.001
Stance time (sec)	0.72 ± 0.02	0.61 ± 0.02	<0.001
H.O & H.S time (sec)	0.02 ± 0.01	0.13 ± 0.01	<0.001
Step length (cm)	50.19 ± 1.62	61.93 ± 1.62	<0.001
Stride length (cm)	100.86 ± 3.23	124.48 ± 3.23	<0.001
Step extremity ratio	0.6 ± 0.02	0.75 ± 0.02	<0.001
Base of support (cm)	12.28 ± 0.64	8.7 ± 0.64	<0.001

*CK, creatine kinase; 6MWT, 6 min walking test; FAP, functional ambulation profile; H.O & H.S time (sec), heel off and heel strike time*.

### Lowe Limb Muscle MRI Findings

**Figure 2:**
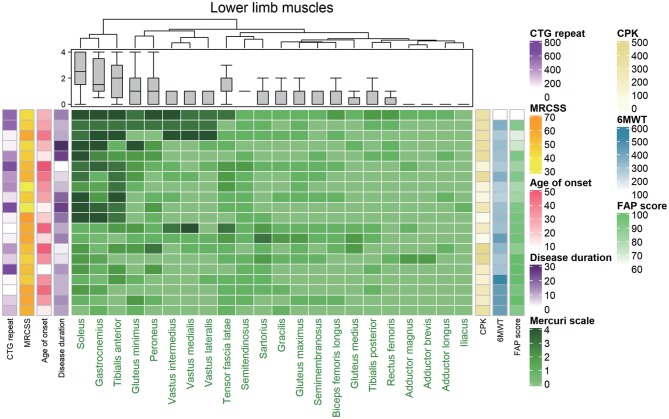
Hierarchically clustered heat map of Mercuri scale ratings from magnetic resonance (MR) images from 20 patients with DM1. The map showed that the included patients could be divided into two distinct groups with mild fatty replacement and with moderate/severe fatty replacement. Each low corresponds to one patient, and they are hierarchically clustered based solely on MR imaging data (using the Mercuri scale). Each column in the heat map corresponds to one muscle, in descending order from the most to least degenerated. A gray-green-midnight green gradient in the heat map indicates increasing fatty substitution (legend at the bottom). The five columns at the left of the heat map denote individual clinical features (not used in the hierarchical clustering algorithm): CTG repeat, CPK, MRCSS, age of onset, and duration of disease, color-coded as indicated in the legend on the right. The three columns at the right of the heat map denote CPK, 6MWT, and FAP score (not used in the hierarchical clustering algorithm) individual clinical features (not used in the hierarchical clustering algorithm), color-coded as indicated in the legend on the right. MRCSS, Medical Research Council sum score; 6MWT, 6 minute walk test.

We evaluated the gluteus maximus, gluteus medius, gluteus minimus, iliacus, sartorius, gracilis, tensor fasciae lata, vastus medialis, vastus lateralis, rectus femoris, adductor magnus, semimembranosus, semitendinosus, long head of biceps femoris, tibialis anterior, peroneus longus, tibialis posterior, soleus, and gastrocnemius muscles. Unsupervised hierarchical clustering of the Mercuri scale rating showed that characteristic fatty replacement patterns on T1-weighted imaging depended on the severity of fatty infiltration (Figure 2). As expected, the hierarchical cluster analysis shows that the muscles in the proximal lower extremities were less affected than those of distal lower extremities. In the distal lower extremity, the soleus, medial gastrocnemius, and tibialis anterior muscles were most severely affected in descending order, whereas the tibialis posterior was least affected (Figure 2). This finding is consistent with the previous reports that crural muscles were more severely affected than proximal leg muscles (22–24). Additionally the cluster analysis well-correlated among the crural muscles (soleus, gastrocnemius, and tibialis anterior) with 6MWT and FAP scores while MRCSS, age of onset, disease duration and CTG repeat lengths showed no correlations. The gastrocnemius muscle was degenerated in all DM1 patients suggesting that the gastrocnemius muscle degenerated earliest and most severely.

### Relationship Between Gait Parameters (FAP Score) and Lower Limb Muscle MRI

We compared individual muscles evaluated by MRI acquired in the lower extremity with the FAP score through the GAIRite apparatus and 6MWT. The grade of Mercuri score of tibialis anterior, soleus, and gastrocnemius muscle were negatively correlated with both FAP and 6MWT (Table 3). Vastus lateralis was the only proximal muscle that showed a correlation with FAP (Table 3). Furthermore, FAP score and 6MWT were significantly correlated (*r* = 0.46, *p* < 0.05).

**Table 3:** Correlation between Mercuri score and gait parameters in DM 1 patients.

	**Gluteus maximus**	**Gluteus medimus**	**Gluteus minimus**	**Iliacus**	**Sartorius**	**Gracilis**	**Tensor facia latae**	**Vastus medialis**	**Vastus lateralis**	**Rectus femoris**	**Adductor magnus**	**Semimembranosus**	**Semitendinosus**	**Long head of biceps femoris**	**Peroneus longus**	**Tibialis posterior**	**Gastrocnemius**	**Tibialis anterior**	**Soleus**
***FAP***	*−0.18*	*−0.18*	*−0.33*	*0,04*	*0.34*	*−0.1*	*−0.26*	*−0.28*	−0.46^*^	*−0.24*	*0*	*0.12*	*0.18*	*−0.29*	*−0.21*	*0.21*	−0.61^*^	−0.62^*^	−0.53^*^
***6MWT***	*−0.01*	*0.22*	*0.15*	*−0.12*	*0.11*	*−0.13*	*0.07*	*0.16*	*−0.19*	*−0.36*	*−0.36*	*−0.24*	*−0.31*	*−0.05*	*−0.23*	*0.05*	−0.62^*^	−0.52^*^	−0.69^*^
Step count																	0.55^*^	0.67^*^	0.6^*^
Velocity (m/sec)																	−0.49^*^	−0.75^*^	−0.46^*^
Cadence (steps/min)																	−0.31	−0.51^*^	−0.19
Step time																	0.3	0.5^*^	0.18
Stride time																	0.3	0.51^*^	0.18
Stance time																	0.33	0.6^*^	0.23
H.O-H.S time																	−0.65^*^	−0.73^*^	−0.67^*^
Step length																	−0.54^*^	−0.63^*^	−0.63^*^
Stride length																	−0.55^*^	−0.69^*^	−0.59^*^
Step extremity ratio																	−0.67^*^	−0.69^*^	−0.68^*^
Base of support																	0.52^*^	0.46^*^	0.33
Toe degree(°)																	0.52^*^	0.4	0.49^*^

* *p < .05., H.O-H.S time, Heel off-Heel strike time. FAP, Functional Ambulation Profile; 6MWT, 6 minute walk test*.

To clarify how the degeneration of each distal limb muscle that play an important role in ankle weakness that is often seen in DM1, correlation analysis between each Mercuri score of tibialis anterior, soleus, and gastrocnemius muscles were evaluated. These muscle groups were compared with the gait parameters including FAP scores (Table 3). The grade of muscle degeneration in tibialis anterior correlated with all spatio-temporal gait parameters. It contributed the most to slow gait velocity of gastrocnemius and soleus (*r* = −0.75 for tibialis anterior, *r* = −0.49 for gastrocnemius, *r* = −0.46 for soleus, respectively). The degeneration of gastrocnemius had the greatest impact on wide base of support, implying that the gastrocnemius was related with wide base gait and may be related to postural instability. Degeneration of all three distal muscles had a significant impact on gait parameters, higher step count, lower cadence, short heel off-heel on strike time, short step length, short stride length, and small step extremity ratio (Table 3).

### Relationship Among FAP Score, Crural Muscles and 6MWT

**Figure 3:**
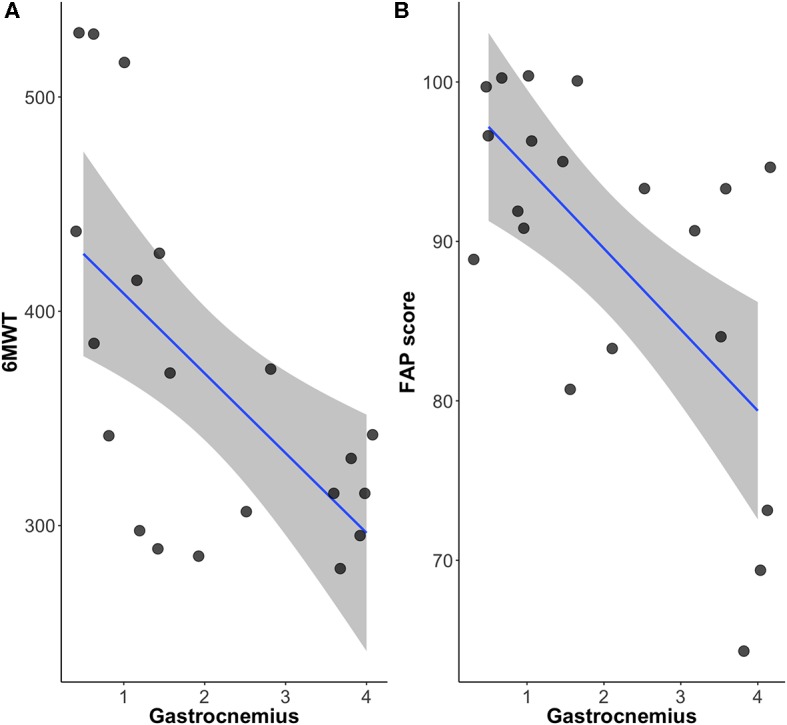
The relationship among gastrocnemius, **(A)** 6 minute walk test and **(B)** FAP scores.

The FAP score that reflects overall functional gait function, correlated with the 6MWT with statistical significance (*r* = 0.46, *p* < 0.05). The Mercuri scale of the crural muscles including tibialis anterior, soleus and gastrocnemius muscles (*r* = −0.52, *p* < 0.05; *r* = −0.69, *p* < 0.05; *r* = −0.62, *p* < 0.01) also correlated well with the 6MWT. Among them, gastrocnemius muscle was the only muscle that significantly correlated with both 6MWT and FAP scores (*r* = −0.61, *p* < 0.01; *r* = −0.62, *p* < 0.01) (Figure 3). The fatty infiltration of the gastrocnemius and tibialis anterior muscles showed statistically significant correlation to step width that reflects the base of support (*r* = 0.52, *p* = 0.019; *r* = 0.46, *p* = 0.05). Moreover, the gastrocnemius muscle also showed significant correlation to the degree of toe (*r* = 0.52, *p* = 0.018), reflecting the role of gastrocnemius muscle in postural instability.

### Relationship Between FAP Score and Clinical/Laboratory Parameters

We compared clinical characteristics such as age of onset, disease duration, creatinine kinase levels and 6MWT that reflects the overall motor function. We also compared the genetic parameter of CTG repeats as well, with the FAP score. The 6MWT statistically correlated with the FAP score (*r* = 0.464, *p* = 0.039), while clinical parameters including age of onset and disease duration showed no correlation with the FAP score. The FAP did not correlate with laboratory parameter such as creatine kinase or genetic parameter of CTG repeat length.

## Discussion

The aim of this study was to evaluate gait patterns in DM1 patients and in healthy controls using GAITRite in relation to muscle imaging and motor performance. We used muscle MRI and 6MWT that are most commonly used biomarkers in DM1. The fatty degeneration of lower muscles are well-known non-invasive radiologic marker in various muscular dystrophies and 6MWT reflects the overall cardiopulmonary and motor function in different muscular dystrophies. We investigated the potential relationship between gait parameters with emphasis on FAP score with these two biomarkers. To the best of our knowledge, this is the first study to analyze the degree of muscle involvement, as assessed with muscle MRI, and its correlations with FAP scores using GAITRite in DM1 patients.

FAP score focuses on spatiotemporal gait parameters that quantitatively show gait speed and distance in various neurological disease (25, 26). FAP score ranges from 30 to 100 points and it is based on self-selected velocity trial. The parameters include step time, step length to leg length ratio (SL/LL ratio), normalized velocity for each leg, degree of asymmetry for SL/LL ratio between two limbs and dynamic base of support. These scores are automatically calculated via deduction from 100 and the lowest score being 30. FAP scores are most extensively studied in Multiple sclerosis (MS) where FAP score well-correlated with Expanded Disability Status Scale (EDSS) which is most widely used clinical scale in MS (12). Although FAP scores are expressed in quantitative values and it is automatically calculated in association with the parameters as described earlier, one of the most important limitation of FAP score is that is can be over-estimated in patients who can walk at a higher speed and it is not validated in children under age of 12 (24, 25). Our study enrolled DM1 patients with a mean age of 44 and it is well-described through numerous studies that showed impairment of gait along with slower speed and instability. Therefore, FAP score may be a simple and an ideal marker that is more suitable in gait evaluation of DM1.

The spatio-temporal gait parameters revealed short stride length and slow cadence resulted in significantly reduced walking velocity. Furthermore, an increased step width (base of support), increased stance time, and stride time also had an impact on slow walking. Walking speed is a recommended tool to evaluate walking ability, and many studies have reported reference or normative values of gait speed (27, 28). A Swedish study recommended a pedestrian walking speed of 1.4 m/s when constructing signalized intersection (29). Our study indicated that DM1 patients had a walking speed of 0.89 m/s, and they would not be able to cross the street in due time, emphasizing the importance in understanding altered gait patterns in DM1 that affect day to day life of these patients. Finally, the main outcome of FAP score, an overall score that reflect the efficacy of gait was significantly different from that of the control group.

Several quantitative studies assessed gait strategy in DM1 patients using different techniques and markers in small samples (4, 5, 7). Wright et al. used infrared-emitting diodes affixed to bracket the hips, knees, and ankles as an assessment tool for gait of DM1 patients (7). They found an abnormal motion pattern at both the hip and ankle, despite the lack of evidence for proximal muscle weakness, such as that of hip muscles. In particular, they described “foot slap” which was prolonged and increased ankle plantar-flexion motion after heel strike (7). Another study using segmental kinematics analysis coupled with ground reaction force measurements and wireless electromyography also confirmed that plantar-flexion motion was both limited and prolonged after heel-strike (foot slap) (6). These studies uniformly show distal muscle weakness that affect gait patterns in DM1. In accordance to the previous studies, gait parameters in our study showed a shorter heel off-heel on time (the heel on time for the next foot minus the heel off time for the current foot), indicating that their plantar flexion activity was prolonged after heel strike. These findings can be consolidated by our muscle MRI findings that illustrated a significantly severe involvement of the gastrocnemius, soleus, and tibialis anterior muscles. Interestingly these crural muscles significantly correlated with the FAP score as well as 6MWT. Moreover, we observed that the gastrocnemius was the most frequently degenerated muscle and showed degeneration earlier than the tibialis anterior muscle. These findings are in line with former MRI reports demonstrating that in DM1, the gastrocnemius muscles were predominantly affected (23, 29). We speculate that the gastrocnemius muscles had the most influence on base of support (step width) and therefore showed a significant correlation with various gait parameters (step count, velocity, heel off-heel on strike time, step length, stride length, and step extremity ratio) (Table 3) in our study. These findings may be partly explained by the “posture first” strategy which DM1 patients chose in order to preserve stability (30). In accordance to these findings, we also observed the importance of gastrocnemius in gait abnormalities in DM1 where it statistically correlated with both FAP score and 6MWT.

The tibialis anterior muscle contributed the most to slow velocity of gait and correlated with all spatio-temporal gait parameters (Table 3). These findings suggest that ankle dorsiflexor weakness influenced all gait components (prolonged stride time, shorter step, prolonged heel off time, wide step, and small cadence) and finally resulted in slow gait speed. In summary, significant degeneration of ankle plantar flexors may produce postural instability, while weakness of dorsal flexors is responsible for the reduction in gait speed.

The novelty of our study is that we found a significant correlation among the FAP score, 6MWT and fatty infiltration in the crural muscles and that these parameters seem to play a synchronous role in understanding impairment of gait in DM1.

Our study has several limitations. First, the small number of DM1 patients was not enough to meet statistical significance, and may not reflect the entire range of alterations associated with DM1. However, this study may be relevant to a substantial portion of ambulatory patients with mild to moderate DM1. Second, this was a cross-sectional study with DM1 patients; as such, longitudinal follow up of DM1 patients is required to investigate the progression of gait abnormalities in conjunction with changes in muscle strength, muscle degeneration, and postural control. Finally, in addition to muscle involvement, cognition, sensory, visual, and hearing symptoms could contribute to gait impairments in DM1 patients. Further studies focusing on central nervous connectivity for deeper understanding of gait performance will be necessary. These data will help to determine whether systemic gait analysis using GAITRite may be used to (1) identify specific gait impairments prior to the occurrence of foot drop in DM1 patients, (2) indicate the need for early intervention, (3) help to tailor the management of DM1 patients, and (4) gait parameters such as FAP may be used as a valuable non-invasive tool in evaluating progression as well as a marker of treatment.

## Conclusion

All spatio-temporal parameters were impaired in DM1 Radiologically most affected muscles were tibialis anterior and gastrocnemius muscles and these muscles significantly influenced FAP score in DM1. Moreover, the gastrocnemius seem to influence postural instability, while tibialis anterior seem to be related to reduction in gait speed based on the result of our GAITRite analysis. Lastly, FAP score well-correlated with the 6MWT as well as fatty degenerative change in the muscle MRI. Therefore, FAP score may be used as a surrogate marker of lower limb motor function and with more longitudinal studies, the use can be extended as a therapeutic marker in near future clinical trials in DM1.

## Data Availability Statement

The raw data supporting the conclusions of this article will be made available by the authors, without undue reservation, to any qualified researcher.

## Ethics Statement

The studies involving human participants were reviewed and approved by Institutional review board of Kyungpook National University Chilgok hospital. The patients/participants provided their written informed consent to participate in this study.

## Author Contributions

SK and Y-HL analyzed and interpreted data. DP, KK, and H-WL contributed to conducting the study in patients. J-SP created the concept, interpreted, and supervised the study.

## Conflict of Interest

The authors declare that the research was conducted in the absence of any commercial or financial relationships that could be construed as a potential conflict of interest.
